# Network Pharmacology Integrated Molecular Docking to Explore the Mechanism of Blister Beetle Therapy for Lung Adenocarcinoma

**DOI:** 10.1155/2022/1892384

**Published:** 2022-07-14

**Authors:** Shoujun Deng, Ying Mao, Heng Li, Gaofeng Li

**Affiliations:** ^1^Department of Thoracic Surgery II, The Third Affiliated Hospital of Kunming Medical University, Yunnan Cancer Hospital, Kunming 650118, China; ^2^Department of Thyroid Breast Surgery, The Second Affiliated Hospital of Kunming Medical University, Kunming 650101, China

## Abstract

Lung adenocarcinoma (LUAD) is one of the major causes of cancer death in the world. Studies show that the effective anticancer component in blister beetles is cantharidin, which can improve chemotherapy efficacy, median survival, and prognosis of LUAD. However, the antitumor mechanism of blister beetles has not been fully clarified. This study aimed to identify the key targets of the treatment of LUAD by blister beetles based on the principle of network pharmacology. An integrated approach including network pharmacology and a molecular docking technique was conducted, which mainly comprises target prediction, weighted gene correlation network analysis (WGCNA) analysis, network construction, gene ontology, and pathway enrichment analysis. 35 key targets were obtained and significantly associated with response to external stimuli, collagen binding, cyclin binding, organic acid binding, pyruvate metabolism, glycolysis, and amino acid biosynthesis pathways. Both LASSO regression and the RF model had a high predictive ability, and 9 candidate genes were screened, among which BIRC5 and PLK1 were the key targets for the treatment of LUAD by using blister beetles and showed significant survival significance. Cantharidin exerts its antitumor effects through 8 targets in 32 pathways, while BIRC5 and PLK1 have obvious survival significance.

## 1. Introduction

Lung cancer is the most common malignant tumor in clinical practice, and its mortality and morbidity rank first in malignant tumors [1]. Moreover, the incidence has been increasing in recent years. According to data released by China's National Cancer Center in 2019, there were about 787,000 new cases of lung cancer and 631,000 deaths in China in 2015. Lung cancer is roughly divided into two categories: non-small-cell lung cancer (NSCLC) and small-cell lung cancer (SCLC). Non-small-cell lung cancer (NSCLC) accounts for about 80% of all lung cancers, mainly including large cell carcinoma, squamous cell carcinoma, and adenocarcinoma [2–4]. Chemotherapy is currently one of the main methods for the treatment of advanced lung adenocarcinoma, but the quality of life of patients has not been significantly improved due to the available drug resistance of tumor cells and severe adverse reactions of chemotherapy drugs [5, 6]. Therefore, it is of great significance to study the molecular mechanism of the occurrence and progression of lung cancer and to find new therapeutic methods and strategies for the clinical diagnosis and treatment of lung cancer.

The blister beetle is a famous Chinese medicine and has been demonstrated in vitro to have significant antitumor effects in cancer cells such as lung, breast, gastric, hepatocellular, and ovarian cancers [7–12]. In China, drugs from blister beetles include Aidi injection (an injection mainly composed of blister beetles), compound cantharidin capsule, sodium cantharidate vitamin B6 injection, and methylcantharidine tablets, which are used in clinical antitumor therapy [13]. Studies show that the effective anticancer component in blister beetles is cantharidin, which has no obvious immunosuppressant effect on the body while inhibiting tumor cells, including cantharidin and its dimethycantharidin, sodium cantharidate, and sodium methyl cantharidate [14, 15]. Clinical practice has proved that the chemotherapy efficacy, median survival, and prognosis of lung cancer patients are improved by drugs derived from blister beetles. For example, compared with platinum-based chemotherapy alone, the clinical efficacy of stage IIIB/IV NSCLC patients was improved by Aidi injection combined with platinum-based chemotherapy, including reduced chemotherapeutic toxicity [1]. In vitro studies have shown that cantharidin (CTD) inhibits cell growth and migration and promotes autophagy and apoptosis by inhibiting the PI3K/Akt/mTOR signaling pathway in non-small-cell lung cancer [16]. Although these studies reported part of the molecular mechanism of cantharidin against tumors, the antitumor mechanism of blister beetles has not been fully elucidated due to the multiactive component, multi-target, and multipathway nature of herbal medicine.

Nowadays, network pharmacology integrates network biology and polypharmacology on the basis of existing databases to provide a new approach to explore the mechanisms and synergistic effect of TCM formulas for the treatments of diseases [17–19]. For example, Li et al. [20] combined network pharmacology, machine learning, and molecular dynamics simulation methods to identify two key genes, insulin-like growth factor 1 receptor (IGF1R) and insulin receptor (IR), and three potential longevity-related herbs from established TCM databases [20]. Tang et al. [21] integrated network pharmacology with molecular docking to unravel the active compounds and potential mechanism of the simiao pill in treating rheumatoid arthritis [21]. Many studies attempting to combine network science with ancient Chinese medicine to study multiple molecular mechanisms have been successful [22–24].

In order to systematically explore the targets of blister beetles in inhibiting LUAD, we integrated complex pharmacology and molecular docking experiments based on four databases to obtain the key chemical components and their targets, which not only provided a new direction for the mechanism of action of LUAD inhibition by blister beetles, but also identified clinically relevant biomarkers that could be used to monitor the treatment of blister beetles.

## 2. Materials and Methods

### 2.1. Weighted Gene Correlation Network Analysis

TCGA database (https://portal.gdc.cancer.gov/) was used to download transcriptomic data of TCGA-LUAD. In this study, WCGNA was used to screen for key modules associated with LUAD [25]. TCGA mRNA expression data profiles were normalized using the “normalizeBetweenArrays” function of the *R* package. The coexpression network of genes with mean expression values greater than 1 in LUAD samples (*n* = 535) and normal samples (*n* = 59) was used to filter out key modules and genes associated with LUAD. First, the samples were clustered and the outlier samples were deleted to ensure the accuracy of the subsequent analysis. Then, the soft threshold is determined and the coexpression network is constructed by using the dynamic tree cutting algorithm. The minimum number of genes per gene module was set to 50 and MEDissThres was set to 0.2. The Limma package (version 3.46.0) [26] was used to compare the difference in gene expression levels between the LUAD/normal sample groups (log2*FC*/≥ 1, *p* < 0.05). Ggplot2 (version 3.3.3) was used to map volcano plots to show differentially expressed genes, The VennDiagram package (Version 1.6.20) was used for differentially expressed genes between LUAD and the control with intersection of key module genes associated with LUAD obtained by WGCNA. The TCMSP (https://tcmspw.com/tcmsp.php) database and the DrugBank (https://go.drugbank.com/) database were used to obtain the active ingredients of blister beetles and their drug targets; the Genecards (https://www.genecards.org/) database was used to predict disease targets in LUAD, retaining only genes with a category of protein coding (category = protein coding and relevance score ≥1). The VennDiagram package was used to intersect key module genes obtained from WGCNA with drug targets and LUAD disease targets from the database to screen for key targets.

### 2.2. Gene Ontology Functional Enrichment Analysis and Kyoto Encyclopedia of Gene and Genomes Analysis

GO stands for Gene Ontology, which is an international standard classification system for gene function. It aims to establish a linguistic vocabulary standard for qualifying and describing gene and protein functions that are applicable to various species. The GO system consists of three components: biological process (BP), molecular functions (MF), and cellular components (CC). Using the GO database, we can obtain what our target genes are mainly related to at the CC, MF, and BP levels. Enrichment analysis of KEGG and GO databases was used to find common functions and related pathways of a large number of genes within the module key genes. In this study, the VennDiagram package was used to obtain key targets by taking intersections of differentially expressed key module genes, drug targets, and LUAD disease targets. The clusterProfiler package [27] was used for GO and KEGG enrichment analysis (*P* < 0.05 and COUNTS ≥2). The enrichplot package (Version 1.10.2) was used to draw bar and bubble graphs to show GO enrichment and KEGG pathway enrichment results.

### 2.3. Network Construction

The key targets of GO_BP, GO_CC, and GO_MF were presented to construct a key target-function regulatory network. The top 50 pathways of KEGG and their corresponding key targets were presented to construct a key target-pathway regulatory network map and were visualized using Cytoscape (version 3.8.2) [28]. To investigate whether there are interactions between key targets, the STRING (https://string-db.org) website was used to construct a PPI network map of 35 key targets with confidence = 0.4 and remove discrete proteins.

### 2.4. LUAD Diagnostic Model Construction

The LASSO regression model and RF algorithm were used to construct a diagnostic model to determine the predictive power of the model constructed based on candidate genes for LUAD. The LASSO analysis tool is a glmnet package (Version 4.1-1) [29]. All TCGA samples (*n* = 594) were used as the training set, and GSE10072 was used as the verification set (*n* = 107) to construct and verify the diagnostic model. The pROC (Version 1.17.0.1) package is used to draw the receiver operating characteristic curve (ROC) of the training set and verification set, and to calculate the area under curve (AUC) value. The higher the AUC value is, the more accurate the prediction is. When the RF algorithm was used to construct the optimal diagnostic model, the expression values of each sample of 35 key targets in TCGA database were combined with the grouping information of the samples, and the RF model was constructed by using the Caret package (Version 6.0-86). Then, the EXPLAIN function of the DALEX package (Version 2.3.0) was used for explanatory analysis of the RF model, and the plot function was used to visualize the distribution of model representation; then, the cumulative residual distribution and boxplot distribution were drawn.

### 2.5. Gene Expression Analysis

The VennDiagram package (Version 1.6.20) was used to take the intersection of the genes obtained by LASSO and the RF algorithm. The ggpubr R package (Version 0.4.0) and the ggplot2 package were used for scatter plotting and visualization to study the expression of nine biomarkers in LUAD samples and normal samples. The test was a rank sum test.

### 2.6. Construction of a Pharmacological Regulatory Network Map and Molecular Docking

The active components corresponding to 9 biomarkers were extracted to construct the drug-active component-key target gene network map, and Cytoscape was used for visual analysis. The protein structure of the key target was obtained from the PDB (https://www1.rcsb.org/) database, and the small molecules and water molecules were removed. Molecular docking is used to determine the presence of interactions between biomarkers and molecules, and to predict their binding patterns and affinities. The AutoDock tools (version 1.5.6) were used to complete protein hydrogenation and charge calculation. The active ingredient structures were downloaded from the PubChem (https://pubchem.ncbi.nlm.nih.gov/) database. Charge balanced and rotatable bond checking for small molecules was performed using AutoDock tools. Then, AutoDock Vina was used to calculate the receptor-ligand docking according to the range of docking boxes selected by the receptor-active center. The structure with the lowest binding free energy in the output results was selected. Finally, PyMol (Version 2.5) software is used for visualization and beautification.

### 2.7. Function Prediction, Prognostic Analysis, and Clinical Correlation Analysis of Biomarkers

GO and KEGG were used for functional and pathway analysis of biomarkers. Kaplan–Meier survival analysis of the biomarkers was performed using *R* language's survival (Version 3.2-3) and survMiner (Version 0.4.8) packages, and *P* < 0.05 indicated that the genes had a significant survival significance. Subsequently, the correlation between biomarkers and clinical factors (age, sex, TNM stage, and stage stage) was studied.

## 3. Results

### 3.1. Active Drug Ingredients of Blister Beetles and Corresponding Targets

To obtain key targets for the interaction between blister beetles and LUAD disease, a total of 27 active drug ingredients and 503 drug targets of the blister beetle were obtained from the TCMSP database. Furthermore, 1236 drug targets were obtained from the DrugBank database and 7031 LUAD disease targets were obtained from the GeneCards database. Subsequently, these targets and key module genes were intersected to obtain key targets.

### 3.2. WGCNA Analysis

**Figure 1:**
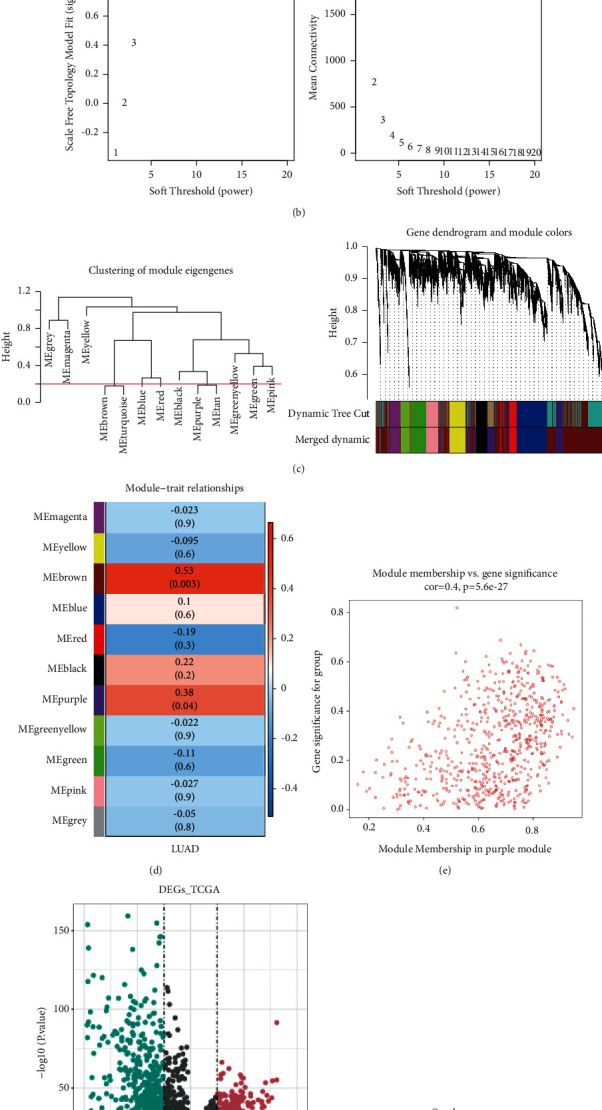
WGCNA analysis. (a) Sample clustering and phenotypic heat map. (b) Filtering of soft thresholds. (c) The merge module. (d) Heat map of the correlation between modules and LUAD. (e) Correlation between modular genes and LUAD. (f) A volcano map of differentially expressed genes between LUAD/normal samples. (g) Screening of key genes in modules. Blue represents the differentially expressed genes between LUAD/control and pink represents the key module genes associated with lung adenocarcinoma acquired by WGCNA.

We clustered all LUAD samples (*n* = 594) and found that there were no outliers, as shown in Figure 1(a). When the ordinate scale-free *R*^2 approaches 0.85 (red line), the soft threshold (*β)* is equal to 5. At this time, the network approaches the scale-free distribution, and mean connectivity also approaches 0. Therefore, we choose the optimal soft threshold (*β*) as 5, as shown in Figure 1(b). A total of 13 modules were obtained, and 11 modules were obtained after module combination, as shown in Figure 1(c). In the module-trait correlation heat map, the MEbrown module had a significant positive correlation with LUAD (Cor = 0.53, *P* = 0.003), and the absolute value of the correlation coefficient is the largest, which is the key module of lung adenocarcinoma, as shown in Figure 1(d). The correlation coefficient between the MEbrown gene and module traits was 0.67 (*P* < 0.05), as shown in Figure 1(e), and there were 2894 genes in the MEbrown module. A total of 1239 differentially expressed genes were found between LUAD and normal samples, of which 571 genes were upregulated and 668 genes were downregulated in LUAD samples, as shown in Figure 1(f). The intersection of the differentially expressed genes between LUAD, control, and the key module genes related to LUAD obtained by WGCNA was conducted to obtain 396 differentially expressed key module genes, which will be studied in the future, as shown in Figure 1(g).

### 3.3. Analysis Results of GO and KEGG

**Figure 2:**
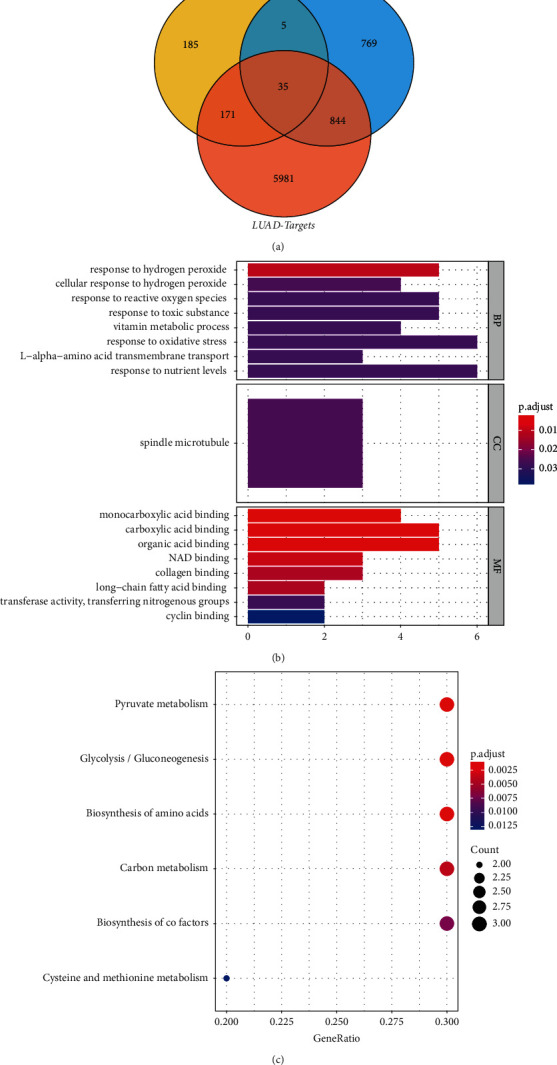
Enrichment analysis results of GO and KEGG of target genes. (a) Venn diagram of the intersection of genes, drug targets, and disease targets. (b) GO enrichment bars of key target genes. (c) KEGG pathway enrichment bubble map of key target genes.

A total of 35 key targets were obtained by taking the intersection of differentially expressed key module genes (*n* = 396), drug targets (*n* = 1653), and LUAD disease targets (*n* = 7031), as shown in Figure 2(a). In terms of biological processes, a total of 50 terms were obtained, and key target genes were significantly associated with responses to toxic substances, oxidative stress, and hydrogen peroxide, including response to nutrient levels, L-alpha-amino acid transmembrane transport, oxidative stress, vitamin metabolic process, toxic substance, reactive oxygen species, cellular response to hydrogen peroxide, and hydrogen peroxide. In terms of molecular functions, 12 terms were obtained, and the key target genes were significantly related to collagen binding, cell cycle protein binding, and organic acid binding functions, including cyclin binding, transferase activity, transferring nitrogenous groups, long-chain fatty acid binding, collagen binding, NAD binding, organic acid binding, carboxylic acid binding, and monocarboxylic acid binding. The results of the study were obtained. In terms of cell composition, a total of 1 term was obtained, and key target genes were significantly associated with spindle microtubules, including spindle microtubules, as shown in Figure 2(b). KEGG functional enrichment analysis of 35 key target genes revealed 6 related pathways, including cysteine and methionine metabolism. Biosynthesis of cofactors, carbon metabolism, biosynthesis of amino acids, glycolysis/gluconeogenesis, and pyruvate metabolism, suggest that the key target genes are related to pyruvate metabolism, glycolysis, and amino acid biosynthesis. Glycolysis and amino acid biosynthesis are shown in Figure 2(c).

### 3.4. Construction of Regulatory Network Diagram

**Figure 3:**
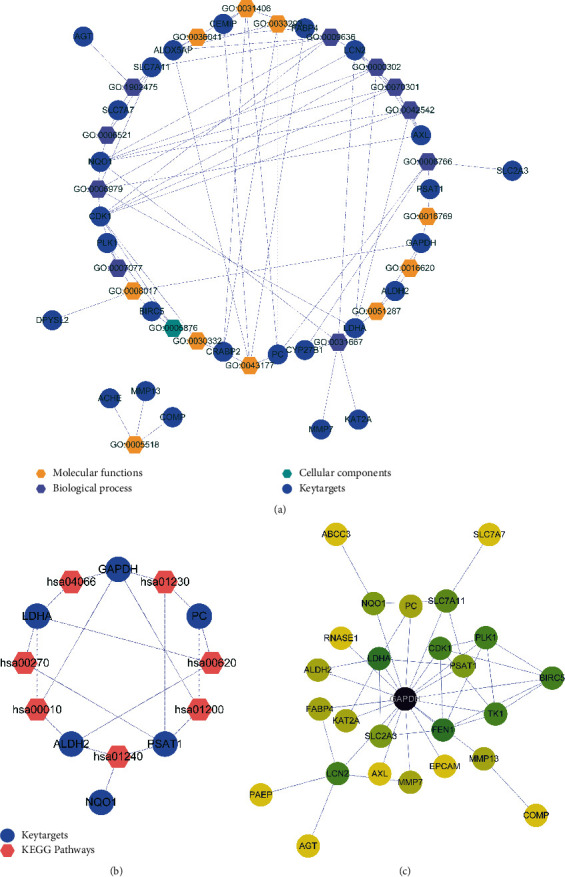
Analysis results of the key target-function/-pathway regulatory network diagram and PPI regulatory network diagram. (a) Regulatory network diagram of the key targets and GO function. The yellow hexagon represents GO_MF terms, the purple hexagon represents GO_BP terms, the green hexagon represents GO_CC Terms, and the blue dot represents the key target. (b) Regulatory network diagram of key targets and pathways. The pink hexagons represent KEGG pathways and the blue dots represent key targets. (c) Protein interaction networks of key targets. The lines represent the interaction between them, the color indicates their degree value; the darker the color, the higher the degree value and the higher the core position.

In the key target-function network diagram, 21 terms, 26 key targets, and 78 relationship pairs were included, as shown Figure 3(a). In the key target-pathway regulatory network map, 50 KEGG pathways, 100 key targets, and 891 relationship pairs were included, as shown in Figure 3(b). The PPI regulatory network diagram of the key targets showed an interaction network of 35 proteins, including 35 nodes and 90 edges, as shown in Figure 3(c).

### 3.5. Construction of the LUAD Diagnostic Model

**Figure 4:**
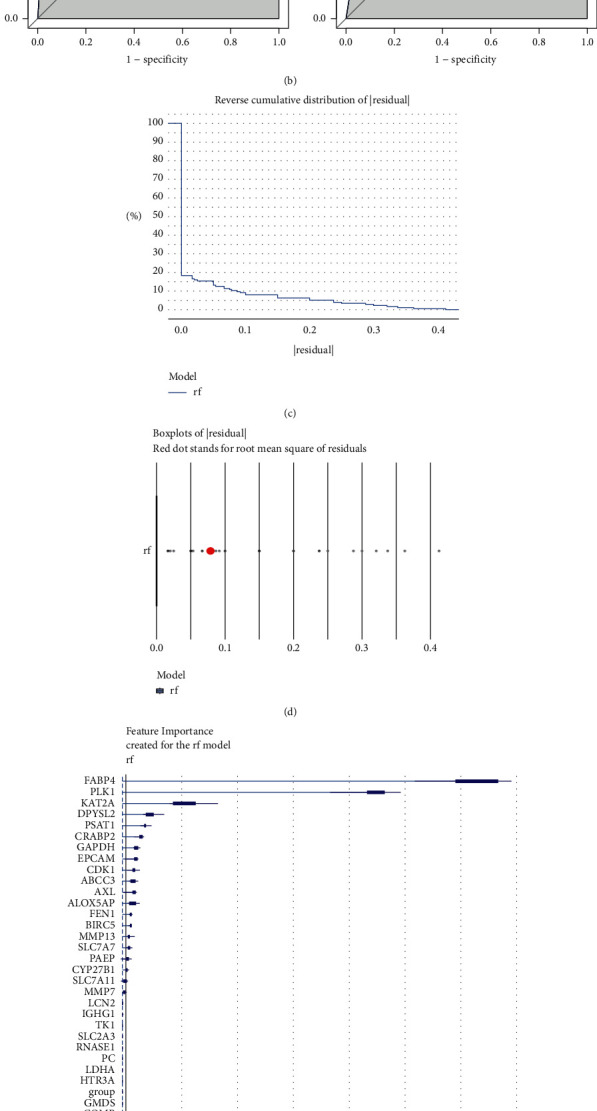
Construction of the LUAD diagnostic model and screening of biomarkers. (a) The characteristic genes were screened by LASSO regression analysis. Deviance on the horizontal axis represents the proportion of residual explained by the model, showing the relationship between the number of characteristic genes and the proportion of residual explained by the model (Dev), and the coefficient of genes on the vertical axis (left). The x-coordinate is log (Lambda), and the y-coordinate represents the error of cross-validation (right). (b) Evaluation and validation of the diagnostic model by the ROC curve. (c, d) The cumulative residual distribution diagram of the sample (left) and the boxplot of the sample residual (right). The curve area indicates the cumulative residual value of the whole sample. The smaller the curve area is, the smaller the cumulative residual value of the sample is. The red dot represents the root mean square of the residual. (e) Importance of genetic variables in the RF models. (f) Evaluation of the RF model by the ROC curve.

In this study, all TCGA samples (*n* = 594) were used as the training set, and GSE10072 was used as the verification set (*n* = 107) to construct and verify the diagnostic model. The graph of the gene coefficient and the error graph of cross validation were obtained by LASSO regression analysis, and a total of 11 characteristic genes were screened, as shown in Figure 4(a). The diagnostic model was constructed from these 11 genes, and the AUCs of the training set and verification set were 0.967 and 0.718, respectively, indicating that the diagnostic model had a high predictive ability for LUAD, as shown in Figure 4(b). When the RF algorithm is used to build the optimal diagnostic model, the sample cumulative residual distribution diagram and boxplot distribution diagram are drawn, as shown in Figures 4(c) and 4(d). In the sample cumulative residual distribution diagram, the smaller the curve area is, the smaller the sample cumulative residual value is. In the boxplot distribution, the smaller the sum of squares of residuals, the better the fitting effect. Different variables have different degrees of relative importance to model prediction, as shown in Figure 4(e). In the RF model, IGHG1, LCN2, MMP7, SLC7A11, CYP27B1, PAEP, SLC7A7, MMP13, BIRC5, FEN1, ALOX5AP, AXL, ABCC3, CDK1, EPCAM, GAPDH, CRABP2, PSAT1, DPYSL2, KAT2A, PLK1, and FABP4 were the predicted values of the response variable (score = 0.07946986) (genes above the response variable were selected as characteristic genes), therefore, these 22 genes were used as diagnostic markers for the next step of analysis in this study. In the RF model, the AUCs of the training and validation sets were 0.985 and 0.978, respectively, indicating that the RF model has high a predictive ability for LUAD, as shown in Figure 4(f).

### 3.6. Genes Expression Analysis

**Figure 5:**
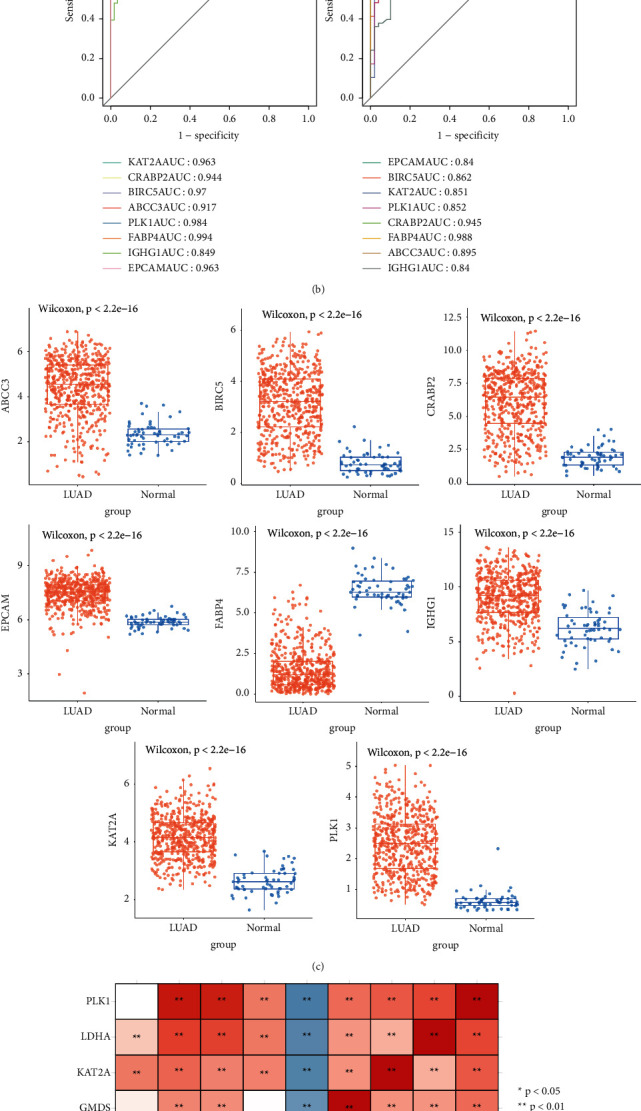
Screening and expression analysis of characteristic genes. (a) Intersection of the LASSO diagnostic model gene and RF model gene. Pink represents the LASSO diagnostic model genes and blue represents the RF model genes. (b) ROC curves for candidate biomarkers. (c) Expression of biomarkers. (d) Correlation of biomarkers. Blue is negative and red is positive.

8 candidate genes (CRABP2, KAT2A, BIRC5, ABCC3, PLK1, FABP4, IGHG1, and EPCAM) were obtained from the intersection of genes obtained by the LASSO model and RF model, as shown in Figure 5(a). In this study, single-gene ROC curves were plotted for eight genes, and AUC values were calculated and validated at GSE10072. The AUC values of all the intersecting genes in the TCGA dataset and the GSE10072 validation set were above 0.8, as shown in Figure 5(b). CRABP2, KAT2A, BIRC5, ABCC3, IGHG1, EPCAM, and PLK1 were upregulated in the LUAD samples, while FABP4 was downregulated in the LUAD samples, as shown in Figure 5(c), and there was a strong correlation between these biomarkers, as shown in Figure 5(d). In addition, the expression and correlation of biomarkers were validated at GSE10072, and the results were consistent with TCGA, as shown in Figure S1.

### 3.7. Construction of a Pharmacological Regulatory Network Map and Molecular Docking

**Figure 6:**
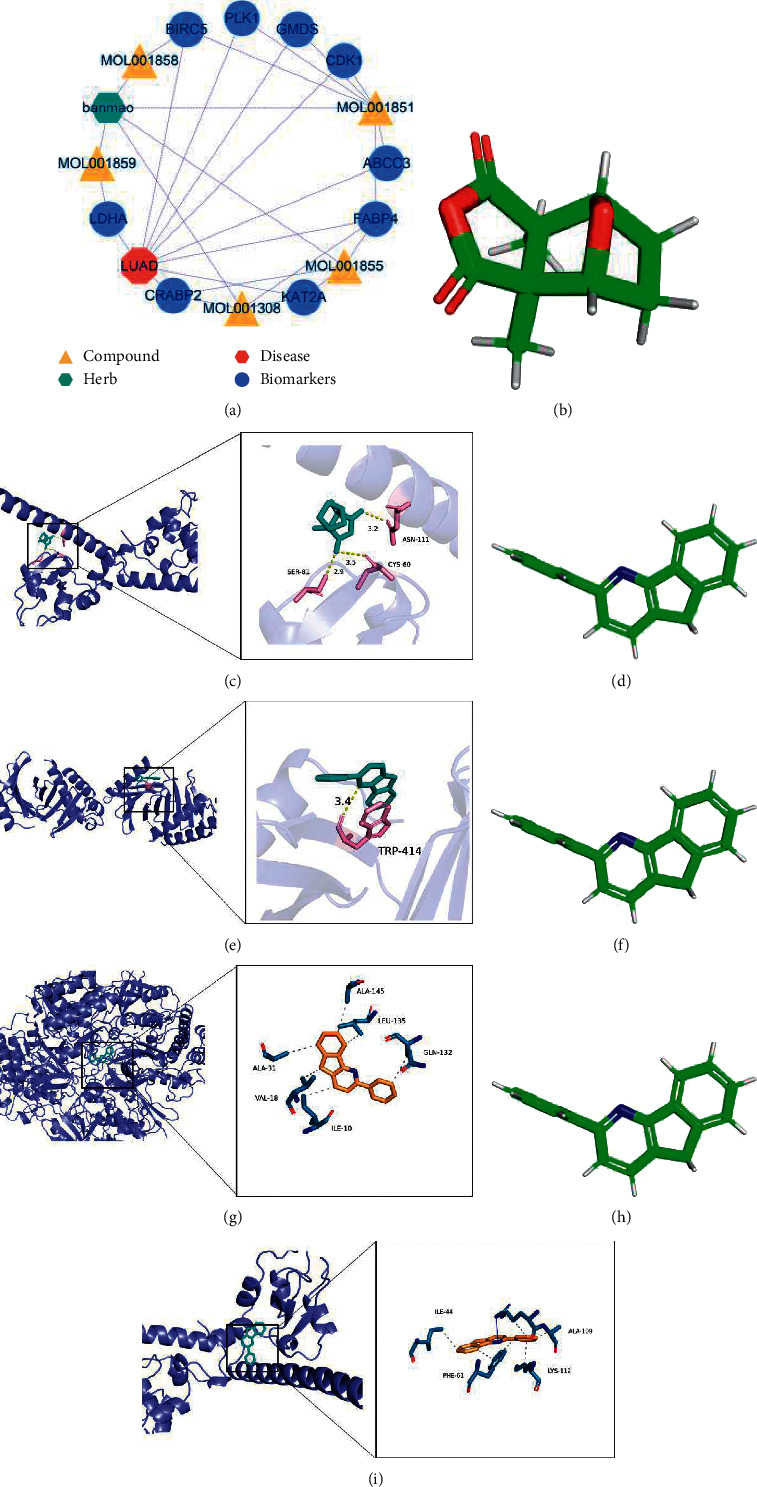
Construction of the pharmacological regulatory network map of Chinese medicine with biomarkers and results of molecular docking. (a) Network diagram of pharmacological regulation of biomarkers in traditional Chinese medicine. (b) 3D conformer structure schematic of cantharidin. (c) Docking result diagram of 3UEH and cantharidin. The green double-ring stick model is the active molecule cantharidin. (d) 3D conformer structure schematic of oleic acid. (e) Docking result diagram of 5TRM and oleic acid. The green double-ring stick model is the active molecule oleic acid. (f) 3D conformer structure schematic of 3-phenyl-4-azafluorene. (g) Docking result diagram of 1Q40 and 3-phenyl-4-azafluorene. The green double-ring stick model is the active molecule 3-phenyl-4-azafluorene. (h) 3D conformer structure schematic of 3-phenyl-4-azafluorene. (i) Docking result diagram of 3UEH and 3-phenyl-4-azafluorene. The green double-ring stick model is the active molecule 3-phenyl-4-azafluorene. Dotted gray/yellow lines represent hydrophobic bonds formed between active ingredients and amino acid residues. Each dotted gray/yellow line represents a hydrophobic bond.

The drug-active ingredient-key target gene network diagram contains 1 drug (blister beetles), 7 drug-active ingredients, 8 biomarkers, and 32 relationship pairs, as shown in Figure 6(a). According to the molecular docking results of the 3D conformer structure of cantharidin, as shown in Figure 6(b), and the crystal structure of BIRC5 with PDB ID 3UEH, there are hydrogen bond interactions between residues of SER-81, ASN-111, CYS-60, and cantharidin molecules. The docking affinity between the active molecule and the protein was −5.2 kcal/mol, as shown in Figure 6(c). According to the molecular docking results of the 3D conformer structure of oleic acid, as shown in Figure 6(d), and the crystal structure of KAT2A with PDB ID 5TRM, there are hydrogen bond interactions between residues of TRP-519, ARG-515, and oleic acid molecules. The docking affinity between the active molecule and the protein was −5.9 kcal/mol, as shown in Figure 6(e). According to the molecular docking results of the 3D conformer structure of 3-Phenyl-4-azafluorene, as shown in Figure 6(f), and the crystal structure of PLK1 with PDB ID 1Q40, the TRP-414 residue has hydrogen bonding interactions with the 3-phenyl-4-azafluorene molecule. The docking affinity between the active molecule and protein was −8.4 kcal/mol, as shown in Figure 6(g). According to the molecular docking results of the 3D conformer structure of 3-phenyl-4-azafluorene, as shown in Figure 6(h), and the crystal structure of BIRC5 with PDB ID 3UEH, there are hydrogen bond interactions between residues of ALA-109, LYS-112, PHE-61, ILE-44, and 3-phenyl-4-azafluorene molecules. The docking affinity between the active molecule and the protein was −6.8 kcal/mol, as shown in Figure 6(i). All have a strong affinity.

### 3.8. Potential Function, Prognostic Significance, and Clinical Relevance of Biomarkers

**Figure 7:**
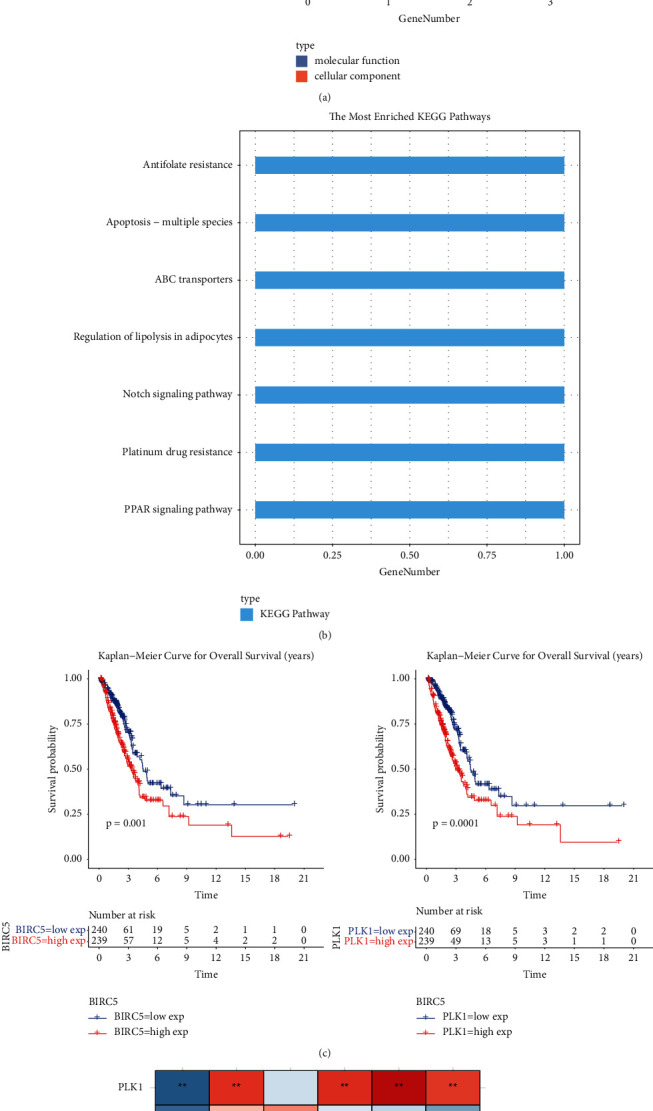
Potential function, prognostic significance, and clinical relevance of biomarkers. (a) Bar chart of GO enrichment results for biomarkers. (b) Bar chart of KEGG enrichment results for biomarkers. (c) Kaplan–Meier survival profile of biomarkers. (d) Heat maps of biomarkers associated with clinical factors.

GO function analysis was performed on the biomarkers, and the biomarkers were mainly enriched into two functional categories of CC and MF (*P* < 0.05 and count ≥ 2). In terms of cell composition, biomarkers were associated with the STAGA complex, dihydro lipid-based dehydrogenase complex, concentrated chromosome, centromere central centromere concentrated chromosome, centromere concentrated spindle microtubules, etc. In terms of molecular function, biomarkers were associated with retinal binding, H4 histone acetyltransferase activity, long-chain fatty acid transporter activity, retinol binding, long-chain fatty acid binding, microtubule binding, organic acid binding, carboxylic acid binding, lipid transporter activity, and monocarboxylic acid binding, as shown in Figure 7(a). Then, KEGG pathway enrichment analysis was performed on the biomarkers (*P* < 0.05 and count ≥ 1), and the biomarkers were mainly enriched into CC and MF functional categories. The results showed that biomarkers were associated with PPAR signaling pathway, platinum resistance, notch signaling pathway, lipolysis regulation of adipocytes, ABC transporter, apoptosis, and antifolic acid resistance, as shown in Figure 7(b). Finally, we explored the prognostic significance of biomarkers. Only BIRC5 and PLK1 were found to have significant survival significance, as shown in Figure 7(c), while the remaining genes were not significant, as shown in Figure S2. We also studied the correlation between biomarkers and clinical factors (age, sex, TNM stage, and stage stage). The results showed that BIRC5 and PLK1 were strongly correlated with clinical factors. In addition, IGHG1 was significantly negatively correlated with *T* staging and stage staging, as shown in Figure 7(d).

## 4. Discussion

In China, blister beetles and their derivatives are used clinically, especially in antitumor therapy, which has been proven to have good effects. However, blister beetles and their derivatives exhibit cardiac, liver, and urinary system toxicity, which limits their clinical application and dose setting [30]. In addition, the application of raw materials of blister beetles leads to high poisoning, including abuse, unreasonable processing, dosage, and accumulation of toxicity [31]. Therefore, the analysis of pharmacodynamic constituents and target of cantharidin, and the molecular mechanism of blister beetles against tumor are helpful to the development of new antitumor drugs.

This study reveals that the main pharmacodynamic components of blister beetles are related to the 8 targets in lung adenocarcinoma. Among them, CRABP2, KAT2A, BIRC5, ABCC3, PLK1, IGHG1, and EPCAM were upregulated in lung adenocarcinoma samples, while FABP4 was downregulated in lung adenocarcinoma samples. Further survival analysis showed that BIRC5 and PLK1 had significant survival significance. The baculoviral inhibitor of apoptosis protein repeat-containing 5 (BIRC5), also known as survivin, is a member of the inhibitor of apoptosis protein (IAP) family and also a target for cancer therapy [32] which exists at the crossroads of many cancer cell signaling networks. Survivin is not only involved in the progression of NSLC, but also related to the development of drug resistance. For instance, reduced survivin expression is a key marker for evaluating prognosis and survival in stage III NSCLC patients who receive platinum-based radiotherapy after surgery [33]. Survivin silencing resensitized A549/VCR cells to vincristine and methotrexate in vitro [34]. Inhibition of survivin expression by the short hairpin RNA expression vector significantly reduced the growth of lung cancer cells in vivo and in vitro [35]. In addition, studies have shown that the polymorphisms of survivin-31 G/C and 9194 A/G are related to the risk of lung cancer. Carriers of CGGC and GCAT haplotypes are more likely to develop lung cancer [36]. These studies suggest that inhibition of survivin is likely to be a major way for cantharidin to inhibit LUAD.

Polo-like kinase 1 (PLK1) is a highly conserved silk/threonine protein kinase that plays an important role in cell cycle regulation and DNA damage repair [37]. Current studies have found that PLK1 is highly expressed in a variety of tumors, including breast cancer, colorectal cancer, endometrial cancer, ovarian cancer, pancreatic cancer, and non-small-cell lung cancer [38]. The PLK1 expression level is related to the degree of malignancy and prognosis of patients [39–42]. Cao et al. [43] found that the downregulation of Mir-886-3p in small-cell lung cancer is closely related to the shorter survival of patients, and Mir-886-3p can inhibit the expression of target genes PLK1 and TGF-*β*1, thus inhibiting the proliferation, migration, and invasion of small-cell lung cancer. Reda et al. [44] found that the application of C-SiPLK1-NP reduced tumor growth and led to prolonged survival. Therefore, PLk1 may be a new prognostic marker and a good target for chemotherapy intervention.

In this study, we obtained the key targets of blister beetles treating LUAD and the biomarkers related to LAUD by using network pharmacology and molecular docking methods based on four databases, indicating that the application of network pharmacology is a scientific and feasible method. However, these results need to be verified in more cell experiments to be better applied in clinical treatment and drug development, including the potential pathway of the Blister beetle antilung cancer activity, the mechanism of action in treating lung cancer, and in vivo experiments to confirm its activity are further needed.

## 5. Conclusions

In conclusion, we screened the key targets of cantharidin for the treatment of LUAD and the biomarkers in LUAD based on network pharmacology and bioinformatics gene analysis. The results revealed that the 7 active compounds exert their antitumor effects via 8 targets in 32 pathways, and BIRC5 and PLK1 had a significant survival significance. This is consistent with the TCM concept of “multiple compounds, multiple targets, and multiple effects.” This provides a systematic view of the potential anticancer mechanism of cantharidin.

## Data Availability

The data that support the findings of this study are openly available in TCMSP (https://tcmspw.com/tcmsp.php); Drug Bank (https://go.drugbank.com/); GeneCards (https://www.genecards.org/); TCGA database (https://portal.gdc.cancer.gov/); STRING (https://string-db.org); PDB database (https://www.rcsb.org/); and PubChem database (https://pubchem.ncbi.nlm.nih.gov/).

## Supplementary Materials

Supplementary Materials 1. Figure S1: expression of biomarkers in GSE10072 dataset. 2. Figure S2: the prognostic significance of the remaining biomarkers.
