# Supplementary figures and images for: Network Pharmacology Integrated Molecular Docking to Explore the Mechanism of Blister Beetle Therapy for Lung Adenocarcinoma
Source: Contrast Media Mol Imaging. 2022 Jul 14;2022:1892384. doi: 10.1155/2022/1892384 (PMC9303499; doi:10.1155/2022/1892384)

**
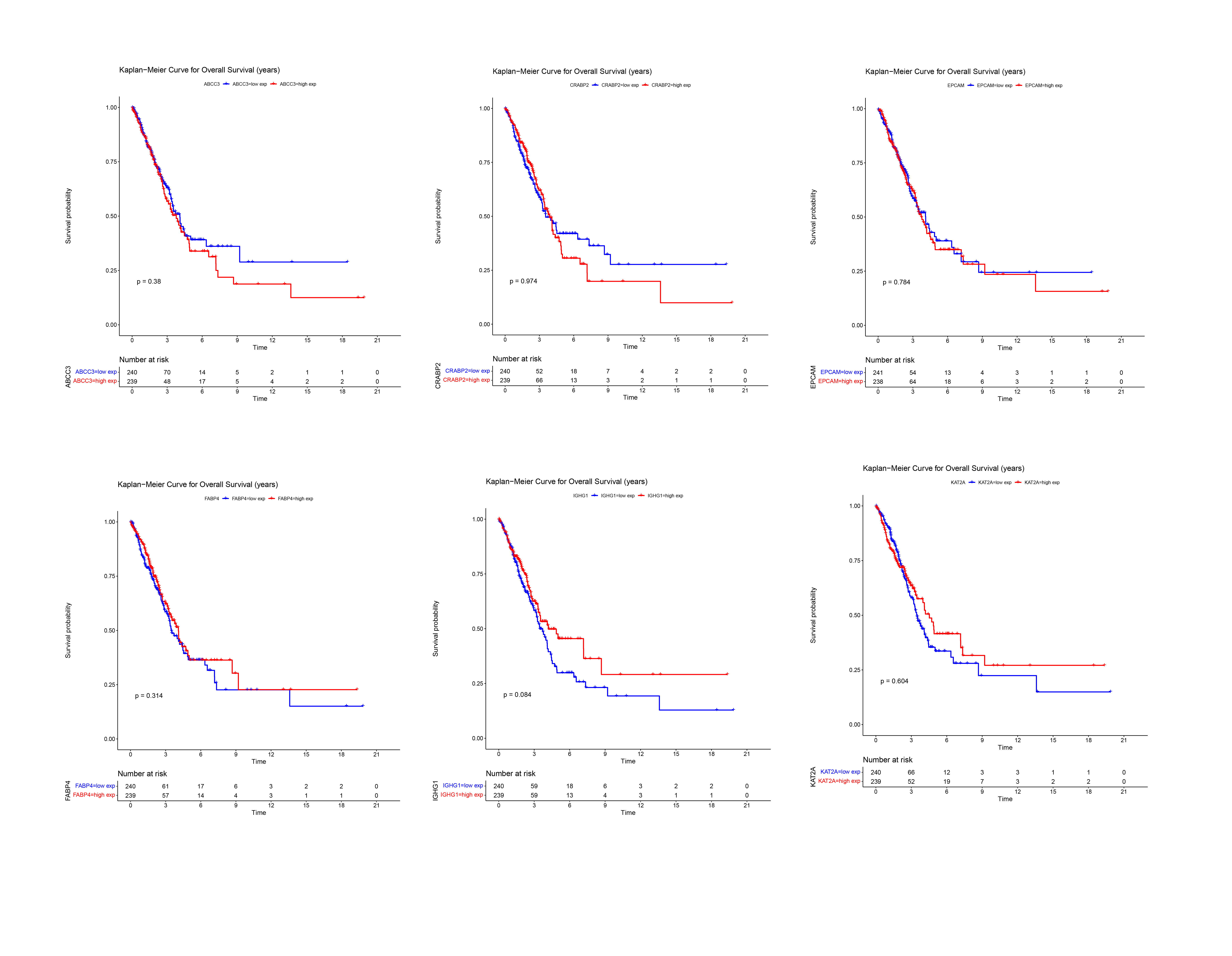
**

Figure S2 The prognostic significance of remaining biomarkers

Supplement: Supplementary Materials — 1. Figure S1: expression of biomarkers in GSE10072 dataset. 2. Figure S2: the prognostic significance of the remaining biomarkers. [file 1892384.f1.zip › 1892384.f1/Figure S2 (2).docx]
